# Sex-specific relationships of inflammatory biomarkers with blood pressure in older adults

**DOI:** 10.1007/s11357-024-01170-8

**Published:** 2024-05-08

**Authors:** Joanna Sulicka-Grodzicka, Barbara Wizner, Tomasz Zdrojewski, Małgorzata Mossakowska, Monika Puzianowska-Kuźnicka, Jerzy Chudek, Andrzej Więcek, Mariusz Korkosz, Elisabetta Caiazzo, Pasquale Maffia, Mateusz Siedlinski, Franz H. Messerli, Tomasz J. Guzik

**Affiliations:** 1 Department of Rheumatology and Immunology, Jagiellonian University Medical College, Jakubowskiego 2, 30-698, Cracow, Poland; 2 Department of Internal Medicine and Gerontology, Jagiellonian University Medical College, Cracow, Poland; 3 Department of Preventive Medicine and Education, Medical University of Gdansk, Gdansk, Poland; 4 Study On Ageing and Longevity, International Institute of Molecular and Cell Biology, Warsaw, Poland; 5 Department of Human Epigenetics, Mossakowski Medical Research Institute, Polish Academy of Sciences, Warsaw, Poland; 6 Department of Geriatrics and Gerontology, Medical Centre of Postgraduate Education, Warsaw, Poland; 7 Department of Internal Medicine and Oncological Chemotherapy, Medical University of Silesia, Katowice, Poland; 8 Department of Nephrology, Transplantation and Internal Medicine Medical, University of Silesia, Katowice, Poland; 9 School of Infection & Immunity, College of Medical, Veterinary and Life Sciences, University of Glasgow, Glasgow, UK; 10 Department of Pharmacy, School of Medicine and Surgery, University of Naples Federico II, Naples, Italy; 11 School of Cardiovascular and Metabolic Health, College of Medical, Veterinary and Life Sciences, University of Glasgow, Glasgow, UK; 12 Department of Internal and Agricultural Medicine, Jagiellonian University Medical College, Cracow, Poland; 13 Medical Genomics Laboratory Omicron, Jagiellonian University Medical College, Cracow, Poland; 14 Department of BioMedical Research, University of Bern, Bern, Switzerland; 15 Centre for Cardiovascular Science, University of Edinburgh, Edinburgh, UK

**Keywords:** Blood pressure, C-reactive protein, Interleukin-6, White blood cells, Older adults

## Abstract

Emerging evidence indicates an association between blood pressure and inflammation, yet this relationship remains unclear in older adults, despite the elevated prevalence of hypertension. We investigated the association between blood pressure, high sensitivity C-reactive protein (hs-CRP), interleukin-6 (IL-6), and white blood cell (WBC) count in a cohort of 3571 older adults aged 65 and above, and 587 middle-aged participants (55–59 years old). In women aged 65 and above, the relationship between inflammatory markers and blood pressure was consistent, with hs-CRP and WBC emerging as predictors of high blood pressure. For hs-CRP, the adjusted odds ratio (OR) was 1.5 (95% CI, 1.07 to 2.10, *P* = 0.02), and for WBC, the adjusted OR was 1.41 (95% CI, 1.02 to 1.94, *P* = 0.04), comparing the highest to the lowest quartiles. In men, only the WBC count was significantly associated with an increased OR for high BP (adjusted OR 1.49, 95% CI, 1.09 to 2.02, *P* = 0.01) across quartiles. Across the entire study population, in a fully adjusted model, all inflammatory markers were modestly associated with blood pressure levels, while the effect of being over 65 years was the most significant predictor of high blood pressure (OR 1.84, 95% CI, 1.50 to 2.25, *P* < 0.001). The link between key inflammation markers and blood pressure in older adults varies by sex and biomarker type and may differ from the relationship observed in younger individuals. These relationships are likely to be affected by factors linked to age.

## Introduction

A possible role of inflammation in hypertension has been demonstrated in several experimental models [1, 2] and human studies [3, 4]. Increased serum levels of C-reactive protein (CRP) and interleukin-6 (IL-6) are associated with high blood pressure and an increased risk of hypertension [5–10]. Thus, although there is evidence of a link between specific inflammatory markers and hypertension, the findings vary and mainly come from studies in younger populations. Research on older adults is scarce, even though more than 60% of individuals over 60 years old experience high blood pressure [11, 12]. C-reactive protein was associated with systolic blood pressure in healthy women aged 60–79 years in the Women’s Heart and Health Study [13]. Additionally, a number of other potentially inflammatory-associated comorbidities have been documented in this group. It seems increasingly important to acknowledge the association between blood pressure and inflammation in an ageing global population, where hypertension is a major contributor to morbidity and mortality. We hypothesized that ageing and comorbidities might confound the association between inflammatory markers and blood pressure in older adults. These relations may also translate to links between inflammation and overall cardiovascular risk associated with hypertension. CRP serum concentration is a useful marker of inflammation and increased risk of cardiovascular complications; furthermore, lower CRP and interleukin-6 (IL-6) concentrations can predict longer survival in older adults [14]. In contrast, IL-6 may have a direct causal role in coronary heart disease (CHD) [15, 16]. Similarly, elevated white blood cell (WBC) count has been identified as a marker of increased CHD risk, and a positive relationship was found between WBC count and systolic and diastolic BP levels [17, 18]. Moreover, a recent Mendelian randomization study in a large UK Biobank population suggested a strong potentially causal relationship between circulating lymphocyte counts and blood pressure [19].

Accordingly, we performed a cross-sectional analysis to evaluate the association between blood pressure levels and prevalent inflammatory biomarkers (concentrations of hs-CRP, IL-6, and WBC counts) in a large cohort of community-dwelling older adults in addition to a group of middle-aged adults, all enrolled in the PolSenior study.

## Methods

The study population included 3571 older adults and a 587 middle-aged reference group, recruited as part of the PolSenior — cross-national, population-based survey, in whom data on inflammatory markers were available. PolSenior was conducted from 2007 to 2009 in a representative sample of the Polish population aged 65 and over (*n* = 4979), with a reference group of 716 middle-aged subjects (55–59 years old). The total number of participants was 5695 (2899 males and 2796 females). The participants were recruited from all administrative regions in Poland using a three-stage stratified, proportional draw. The details of the design and recruitment criteria for the PolSenior survey, as well as descriptions of examination procedures and the structure of the study group, have been reported previously [20]. The sex of participants was defined based on self-report. The PolSenior project was approved by the Bioethics Committee of the Medical University of Silesia in Katowice (KNW-6501–38/I/08). Each subject or their caregiver signed an informed consent form before the enrolment in the study.

### *Chroniccomorbidities*

We estimated the prevalence of 15 chronic conditions, with self-reported data collected through the interview using the standardized questionnaire, which included questions about present health status and history of chronic diseases and hospitalizations. For example, the prevalence of diabetes was determined using self-reported data from an interview survey, where participants answered questions including physician-diagnosed diabetes, without specifying the type. Chronic diseases were grouped into the following categories: cardiovascular (coronary heart disease/myocardial infarction, heart failure, hypertension, arrhythmia, stroke), endocrine (diabetes, thyroid disorders), respiratory (asthma, chronic obstructive pulmonary disease), gastrointestinal (ulcer), chronic kidney disease, osteoporosis, cancer, eye (glaucoma, cataract), neurological (Parkinson’s disease), and mental diseases.

### *Antihypertensivemedications*

The use of antihypertensive medications was based on the assessment of medications taken during the week preceding the examination, regardless of whether the indications were hypertension or other cardiovascular diseases. Polypharmacy was defined as using five or more medications for different clinical indications.

### *Assessmentofblood pressureand anthropometric data*

Blood pressure (BP) measurements were performed three times during the first and second visits to subjects’ homes by trained nurses using validated devices. Each measurement was performed with the participant in a seated position, on the right upper arm, after at least 5 min of rest and at 2-min intervals. High blood pressure (HBP) was defined as average BP values from all BP readings during each visit ≥ 140 mmHg (systolic BP) and/or ≥ 90 mmHg (diastolic BP). Normal blood pressure (NBP) was defined as average BP values from all BP readings during each visit < 140 mmHg (systolic BP) and < 90 mmHg (diastolic BP). Both HBP and NBP groups included participants on antihypertensive treatment and those without current treatment for hypertension. Anthropometric parameters included height and weight. Obesity was defined as BMI ≥ 30 kg/m^2^.

### *Measurement of biochemical parameters*

Venous blood was collected using a vacutainer system and delivered in a cooler to local laboratories within 2 h, where serum and plasma samples were separated and frozen. Blood samples were delivered to the central laboratory for further analysis. Interleukin-6 levels were measured in serum using enzyme-linked immunosorbent assay (R&D System, Minneapolis, MN, USA, the limit of detection — LoD of 0.04 pg/mL), and plasma hs-CRP levels were measured using a high-sensitivity immunoturbidimetric method (Modular PPE, Roche Diagnostics GmBH, Mannheim, Germany, LoD 0.11 mg/L). For this study, participants with leukocyte counts > 10 × 10^9^/L (*n* = 191) and/or hs-CRP > 10 mg/L (*n* = 486), indicating clinically significant inflammation [21, 22], were excluded from further analyses. After the additional exclusion of participants with missing data on inflammatory markers levels (hs-CRP: *n* = 1047; IL-6: *n* = 1188), the study population consisted of 3571 older adults and 587 middle-aged reference groups.

### *Statisticalanalysis*

Quantitative variables are presented as means ± SD or medians (lower and upper quartile), and qualitative variables are presented as numbers and percentages. The Shapiro–Wilk test was used to check if continuous variables follow a normal distribution. Non-normally distributed variables were transformed by natural logarithm (ln). Comparisons between the groups (older adults vs middle-aged subjects) were performed using a two-tailed Student’s *t*-test and the chi-squared test for qualitative variables. Next, we performed two-way factorial ANOVA to compare the main effects of age (two groups: 55–59 years old and ≥ 65 years old) and sex, and the interaction between effects of age and sex on levels of BP and inflammatory markers. The Pearson’s correlation coefficient was calculated to assess the relationship between systolic BP, diastolic BP, and inflammatory markers (ln hs-CRP, ln IL-6, and WBC). A multiple linear regression analysis was run in the group ≥ 65 years old to investigate whether the effects of inflammatory markers on systolic BP level interact with sex. Results are reported as standardized *β*-coefficients and 95% CI. Subsequently, multiple logistic regression was used to estimate odds ratios (95% CI) for HBP according to hs-CRP, IL-6, and WBC, separately for sex-specific quartiles in men and women. Odds ratios were calculated in unadjusted models, models adjusted for age, BMI, smoking, and number of chronic diseases excluding hypertension, and in a fully adjusted model in all participants (*n* = 4158) for the effects of age (ref. middle-aged group), sex (ref. female), number of chronic diseases excluding hypertension, BMI (ref. non-obese), current smoking (ref. non-smoking), and inflammatory markers (WBC, ln hs-CRP, and ln IL-6). The Hosmer–Lemeshow test for the goodness of fit of logistic regression was used. Statistical significance was considered for *P* < 0.05. The statistical analyses were performed with Statistica (data analysis software system), version 13. TIBCO Software Inc. (2017) and SAS 9.4 (SAS Institute Inc., Cary, NC, USA).

## Results

### *Patientscharacteristics*

**Fig. 1:**
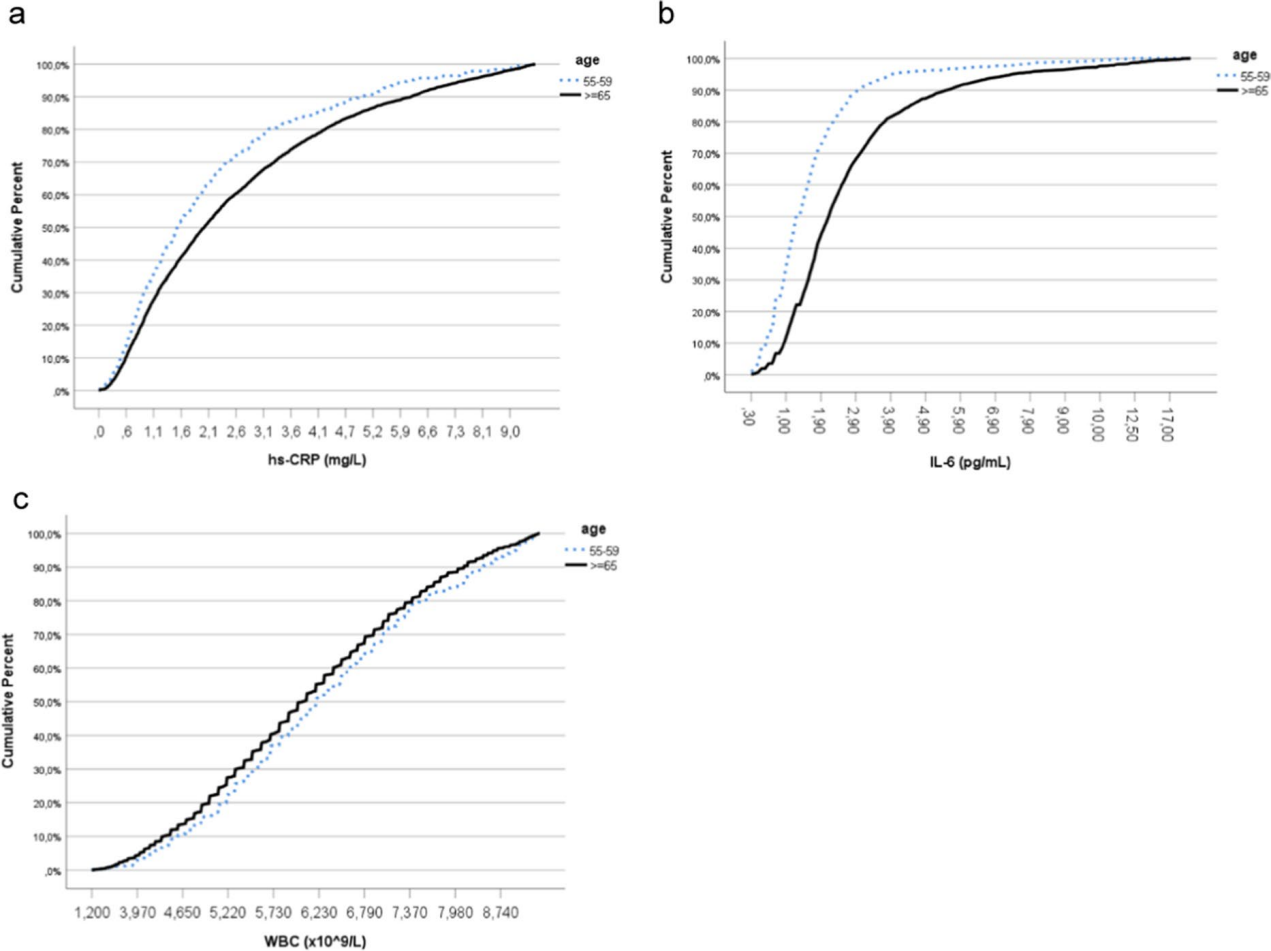
Cumulative percent of hs-CRP, IL-6 levels, and WBC counts in older vs. middle-aged participants. Hs-CRP, high sensitivity *CRP*; IL-6, interleukin 6; WBC, white blood cells

The mean age of 3571 older adults included in the study was 78 ± 9 years, and the 587 middle-aged reference group was 57 ± 1 years. The study group of older adults had a significantly higher prevalence of HBP and male sex than the reference group of middle-aged adults. The prevalence of obesity was comparable in both groups. Almost a third of participants aged 65 and over, and 18.4% of middle-aged adults had three or more chronic diseases (Table 1). Women aged 65 and over had higher BMI than men (28.9 ± 5.4 kg/m^2^ vs. 27.4 ± 4.4 kg/m^2^; *P* < 0.001). Women had a significantly higher concentration of hs-CRP than men in both groups: median 2.1 (1.1–3.8) mg/L vs. 1.9 (0.9–3.6) mg/L in women and men ≥ 65 years old, respectively; *P* = 0.003, and median 1.7 (0.9–3.1) mg/L vs. 1.4 (0.7–2.4) in middle-aged women and men; *P* = 0.002. Men had significantly higher WBC than women in both groups: mean 6.3 ± 1.4 × 10^9^/L vs. 6.1 ± 1.4 × 10^9^/L in men and women ≥ 65 years old, respectively; *P* < 0.001, and 6.5 ± 1.5 × 10^9^/L vs. 6.2 ± 1.4 × 10^9^/L in middle-aged men and women, respectively; *P* = 0.03). The distribution of levels of hs-CRP, IL-6, and WBC count in middle-aged and older participants is shown in Fig. 1.

**Table 1:** Characteristics of the PolSenior study participants

	≥ 65 years old *n* = 3571	55–59 years old *n* = 587	*P*
Age, years	78 (9)	57 (1)	< 0.001
Sex, male, *n* (%)	1834 (51.4)	265 (45.1)	0.005
No. of chronic diseases			
0	634 (17.8)	161 (27.5)	< 0.001
1	907 (25.5)	172 (29.4)	0.023
2	905 (25.4)	145 (24.7)	0.359
3 +	1115 (31.3)	108 (18.4)	< 0.001
Smoking, *n* (%)	297 (8.3)	170 (29)	< 0.001
Obesity, *n* (%)	1081 (31.7)	186 (32.1)	0.87
BMI, kg/m^2^	28.1 (5)	28.2 (4.9)	0.70
WBC, × 10^9^/L	6.2 (1.4)	6.4 (1.4)	0.002
Hs-CRP, mg/L	2 (1–3.7)	1.5 (0.8–2.8)	< 0.001*
IL-6, pg/mL	2.2 (1.4–3.4)	1.3 (0.9–2.1)	< 0.001*
SBP, mmHg	146 (22)	138 (19)	< 0.001
DBP, mmHg	83 (11)	86 (11)	< 0.001
HBP, *n* (%)	2166 (60.8)	277 (47.2)	< 0.001

Data is shown as means (SD), medians (IQR), or numbers (percentages). *Student’s *t*-test for ln-transformed CRP and IL-6 values. *BMI*, body mass index; *WBC*, white blood cells; *hs-CRP*, high-sensitivity C-reactive protein; *IL-6*, interleukin-6; *SBP*, systolic blood pressure; *DBP*, diastolic blood pressure; *HBP*, high blood pressure

Hypertension only, without any other comorbidities, was reported in 30.1% of older participants and in 42.2% of middle-aged individuals. The most prevalent self-reported chronic diseases in older participants were hypertension (57%), eye disorders (35.6%), arrhythmia (33.9%), coronary heart disease (22.2%), and respiratory diseases (17.3%), followed by diabetes (17.1%), while in middle-aged adults, these were hypertension (47%), arrhythmia (25.3%), thyroid disorders (12.6%), mental health diseases (12%), respiratory (10.8%), and coronary heart disease (10.7%).

Participants ≥ 65 years old had a higher prevalence of polypharmacy (55%) than middle-aged individuals (24%), including both prescription and over-the-counter medications (*P* < 0.001). The mean number of medications per day was 5.1 ± 3.5. The prevalence of antihypertensive medications in the high blood pressure group was 65.2%, and in patients with normal blood pressure, it was 61.2%. (*P* = 0.01). Participants were taking ACE inhibitors (37.2%), beta-blockers (33.1%), diuretics (27.4%), calcium channel blockers (15.8%), angiotensin II receptor blockers (7.3%), and alpha-blockers (7.3%).

**Fig. 2:**
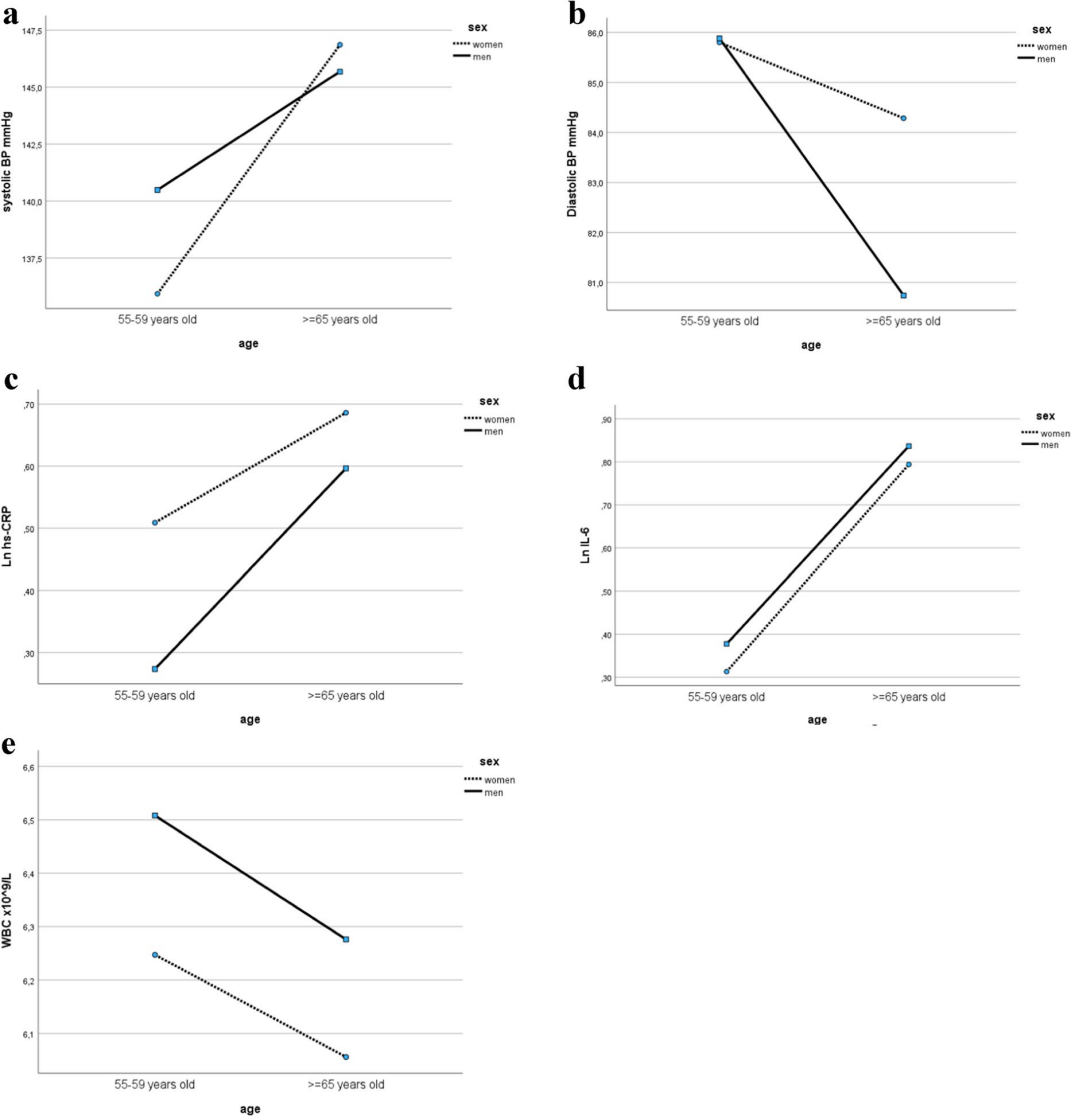
Interaction plots of age and sex effects on the levels of blood pressure (**a**, **b**), hs-CRP (**c**), IL-6 (**d**), and WBC (**e**) counts. Ln, natural logarithm transformed values; BP, blood pressure; hs-CRP, high sensitivity CRP; IL-6, interleukin 6; WBC, white blood cells

We performed factorial analysis (ANOVA) to assess the effects of age (two groups: 55–59 years old and ≥ 65 years old), sex, and their interaction on BP level and inflammatory markers. Age had a statistically significant effect on systolic BP, but the effect of age interacted with sex (*F* = 8.80, *P* = 0.03). Associated with age increase of systolic BP was more expressed in women than in men. A significant difference in systolic BP levels observed between middle-aged men and women disappeared in participants ≥ 65 years old (Fig. 2a). In relation to diastolic BP, we also observed a significant effect of age and sex interaction (Fig. 2b), expressed by a higher decrease of diastolic BP with age in men than in women (*F* = 13.62, *P* < 0.001).

Sex and age independently influenced hs-CRP (*F* = 16.33 and *F* = 38.60 respectively; *P* < 0.001), and WBC (*F* = 14.14 and *F* = 10.95 respectively, *P* < 0.001). There was no interaction effect of age and sex on IL-6, and only age significantly influenced IL-6 (*F* = 236.47, *P* < 0.001) (Fig. 2c–e).

### *BP and systemic inflammation markers in participants* ≥ *65 years old*

The levels of hs-CRP were weakly, positively associated with systolic BP (*r* = 0.07, *P* < 0.001), and diastolic BP (*r* = 0.10, *P* < 0.001). WBC counts were weakly, positively associated with systolic BP (*r* = 0.09, *P* < 0.001) and diastolic BP (*r* = 0.08, *P* < 0.001). IL-6 was weakly, negatively associated with diastolic BP (*r* =  − 0.04, *P* = 0.042) and was not statistically significantly associated with systolic BP. Among inflammatory markers, only IL-6 levels were positively associated with age (*r* = 0.29, *P* < 0.001).

A multiple regression analysis was run in the group ≥ 65 years old to investigate whether the effects of hs-CRP and WBC on systolic BP level change in relation to sex. Hs-CRP was positively related to systolic BP (*β* = 2.49, 95% CI 1.38 to 3.59; *P* < 0.001), and the effect of hs-CRP on systolic BP interacted with sex (*β* =  − 0.76, 95% CI 1.38 to − 0.15; *P* = 0.015). There was a relatively lower increase in systolic BP with increasing hs-CRP in men, than in women. WBC counts independently predicted systolic BP levels (*β* = 1.30 mmHg, 95% CI 0.55 to 2.06; *P* < 0.001).

### *Inflammatory markers in association with high blood pressure in participants* ≥ *65 years old*

The unadjusted OR of high BP for participants (≥ 65 years old overall) in the highest as compared with the lowest quartile for hs-CRP was 1.36 (95% CI, 1.12 to 1.65, *P* = 0.002), and in the highest as compared with the lowest quartile for WBC was 1.42 (95% CI, 1.17 to 1.73, *P* < 0.001). The adjusted for age, BMI, current smoking, and a number of chronic diseases (excluding hypertension) OR for high BP was 1.32 (95% CI, 1.05 to 1.66, *P* = 0.02) and 1.41 (95% CI, 1.13 to 1.76, *P* = 0.002) for highest vs. lowest quartiles of hs-CRP and WBC, respectively. IL-6 was negatively associated with high BP (adjusted OR 0.73, 95% CI, 0.57 to 0.93, *P* = 0.01 for highest compared with lowest quartile).

Next, we analyzed the predictive value of hs-CRP, IL-6, and WBC quartiles for high BP distinctly in men and women ≥ 65 years old in an unadjusted logistic regression model and in an adjusted model. The adjusted OR of high BP for women in the highest as compared with the lowest quartile for CRP was 1.5 (95% CI, 1.07 to 2.10, *P* = 0.02). Other markers significantly associated with the OR of high BP were WBC in women, with adjusted OR 1.41 (95% CI, 1.02 to 1.94, *P* = 0.04) for women in the highest as compared with the lowest quartile, and WBC in men with adjusted OR 1.41 (95% CI, 1.13 to1.76, *P* = 0.01) for the highest as compared with the lowest quartile of this marker in men. IL-6 was a negative predictor of high BP in older women (OR 0.67, 95% CI 0.48 to 0.96, *P* = 0.025) (Table 2).

**Table 2:** Inflammatory markers as predictors of HBP in participants ≥ 65 years old

	Unadjusted model OR (95% CI)	*P*	Adjusted model* OR (95% CI)	*P*
Men				
Hs-CRP (mg/L)				
Q1 (< 0.9)	–		–	
Q2 (≥ 0.9–1.9)	1.06 (0.80–1.41)	0.692	1.01 (0.75–1.35)	0.958
Q3 (≥ 1.9–3.6)	1.01 (0.75–1.35)	0.945	0.93 (0.68–1.25)	0.614
Q4 (≥ 3.6)	1.25 (0.91–1.70)	0.170	1.15 (0.83–1.59)	0.395
IL-6 (pg/mL)				
Q1 (< 1.5)	–		–	
Q2 (≥ 1.5–2.2)	0.94 (0.71–1.26)	0.682	0.99 (0.74–1.32)	0.925
Q3 (≥ 2.2–3.4)	0.85 (0.64–1.14)	0.274	0.93 (0.68–1.25)	0.614
Q4 (≥ 3.4)	0.70 (0.52–0.96)	0.026	0.83 (0.60–1.16)	0.275
WBC (× 10^9^/L)				
Q1 (< 5.2)	–		–	
Q2 (≥ 5.1–6.2)	1.15 (0.87–1.52)	0.317	1.16 (0.87–1.55)	0.304
Q3 (≥ 6.2–7.2)	1.12 (0.84–1.49)	0.454	1.08 (0.80–1.46)	0.600
Q4 (≥ 7.2)	1.52 (1.13–2.05)	0.006	1.49 (1.09–2.02)	0.012
Women				
Hs-CRP (mg/L)				
Q1 (< 1.1)	–		–	
Q2 (≥ 1.1–2.1)	1.25 (0.93–1.68)	0.139	1.20 (0.86–1.64)	0.237
Q3 (≥ 2.1–3.8)	1.43 (1.06–1.93)	0.019	1.47 (1.07–2.01)	0.017
Q4 (≥ 3.8)	1.50 (1.10–2.05)	0.011	1.50 (1.07–2.10)	0.019
IL-6 (pg/mL)				
Q1 (< 1.4)	–		–	
Q2 (≥ 1.4–2.1)	1.04 (0.77–1.39)	0.819	0.96 (0.71–1.31)	0.795
Q3 (≥ 2.1–3.3)	1.07 (0.79–1.45)	0.659	0.96 (0.69–1.33)	0.799
Q4 (≥ 3.3)	0.75 (0.55–1.02)	0.066	0.67 (0.48–0.96)	0.025
WBC (× 10^9^ /L)				
Q1 (< 5.1)	–		–	
Q2 (≥ 5.1–5.9)	0.89 (0.67–1.19)	0.438	0.89 (0.66–1.20)	0.433
Q3 (≥ 5.9–7.0)	0.92 (0.69–1.23)	0.571	0.91 (0.67–1.24)	0.557
Q4 (≥ 7.0)	1.34 (0.99–1.82)	0.062	1.41 (1.02–1.94)	0.040

^*^Model adjusted for the effects of age, body mass index, current smoking, and number of comorbidities excluding hypertension; Dashes indicate reference categories. *WBC*, white blood cells; *hs-CRP*, high sensitivity C-reactive protein; *IL-6*, interleukin-6; *Q*, quartiles

Finally, we estimated the logistic regression model adjusted for age, sex, obesity, current smoking, and a number of chronic diseases (excluding hypertension) in both age groups combined, to assess the association of inflammatory markers with high BP. All inflammatory markers remained modestly associated with high BP; however, the effect of age ≥ 65 years on high BP was the most significant among predictors (OR 1.84; 95% CI, 1.50 to 2.25, *P* < 0.001). The number of chronic diseases was negatively associated with high BP (Table 3).

**Table 3:** Multiple logistic regression model identifying factors associated with high blood pressure among all participants (*n* = 4158)

	OR (95% CI)	*P*
Age		
55–60 years	–	
≥ 65 years	1.84 (1.50–2.25)	< 0.001
Sex		
Women	–	
Men	0.99 (0.86–1.14)	0.910
Obesity		
No	–	
Yes	1.21 (1.04–1.42)	0.015
Current smoking	0.95 (0.76–1.19)	0.659
No of chronic diseases*	0.89 (0.84–0.94)	< 0.001
Hs-CRP^#^	1.12 (1.03–1.22)	0.009
IL-6^#^	0.89 (0.79–0.99)	0.035
WBC	1.10 (1.04–1.16)	< 0.001

^*^Excluding hypertension; ^#^ln-transformed values. Dashes indicate reference categories. *WBC*, white blood cells; *hs-CRP*, high-sensitivity C-reactive protein; *IL-6*, interleukin-6; Hosmer–Lemeshow test statistics *χ*^2^ = 4.55, *P* = 0.81

## Discussion

This study among adults aged 65 and over identified an association between blood pressure levels and serum CRP in women and WBC counts in both men and women and a negative association of IL-6 and blood pressure in women. Although inconsistent among studied markers, and in men and women, the identification of these associations in older adults is of particular importance as it may indicate a role for inflammatory activation in some of the age-associated conditions [23, 24].

To evaluate the strength of our observational results and possibly make a causal inference, we assessed Bradford Hill Criteria [25]. The associations between inflammatory markers and blood pressure in our study were statistically significant, but relatively modest, and accounted for 32% and 41% higher odds for high blood pressure in participants ≥ 65 years old in the upper quartile relative to the first quartile of hs-CRP and WBC, respectively. After adjustment, hs-CRP and WBC remained modestly associated with high blood pressure in all participants; however, the effect of age ≥ 65 years on high BP was still the strongest among predictors.

There is a consistency among some epidemiologic studies of the association between CRP, WBC, and blood pressure levels in different populations, including mostly middle-aged patients. Nevertheless, there is no consistency with regard to sex. Higher CRP was significantly associated with systolic and diastolic blood pressure in young and middle-aged women, but not in men [26, 27]. Women with CRP > 3 mg/L, but not men, had 1.4 times the odds (CI 95%, 1.08 to1.94) of being hypertensive when compared with women with serum CRP levels ≤ 3 mg/l [28]. However, the cause of these sex differences is unclear. Both endothelial cells and vascular smooth muscle cells express estrogen receptors, and estrogen increases the synthesis of NO in blood vessels [29, 30]. Declining levels of estrogen may contribute to maintaining inflammation in older women with atherosclerosis and other comorbidities. Moreover, CRP levels may be more sensitive to fat distribution in women compared with men, and the contribution of adiposity to inflammation may be more relevant in women [31, 32]. Significant differences between men and women in cardiovascular disease biomarkers have been reported. Adipokines and inflammatory markers such as leptin and CRP are higher in women than in men and interestingly less influenced by menopausal or hormone status than platelet and coagulation markers, which supports a potential link between inflammation and CVD preferentially in women [33]. Sex differences occur in both innate and adaptive immune responses, resulting in different susceptibility to various diseases, as women present stronger immune responses than men [34]. Some studies also reported a positive relationship between lymphocyte counts and hypertension [17] and a positive association between neutrophil-to-lymphocyte ratio and the prevalence of hypertension [35]. IL-6 is a pleiotropic cytokine, with both proinflammatory and anti-inflammatory effects. About 25–30% of circulating IL-6 originates in adipose tissue [36, 37]. There are sex differences in adipose tissue distribution, which diminish after menopause. After menopause, women develop more central obesity. Furthermore, in our study, women in the highest IL-6 quartile were significantly older than women in the lowest quartile of IL-6, which may to some extent explain the observed negative relationship of IL-6 with blood pressure level, as blood pressure tends to be lower in octogenarians in comparison to younger subjects. The mechanisms underlying the relationship between IL-6 and blood pressure are likely multifactorial. One should also remember that while IL-6 is generally pro-inflammatory, it may also stimulate some anti-inflammatory responses, especially in certain physiological contexts, which may include older age. Moreover, one cannot exclude that in the context of cardiovascular ageing and hypertension, IL-6 might be involved in compensatory response aimed at preserving vascular homeostasis. These findings emphasize that further studies are required in order to understand the implications of IL-6 in the pathogenesis of hypertension.

Clearly, the association of inflammatory markers with blood pressure is rather not specific and may be confounded by a myriad of factors such as genetic predisposition, diet, body mass index, smoking, medications, comorbidities, and socioeconomic status, as both high blood pressure and inflammation share multiple causes and multiple consequences. Establishing a temporal relationship of the association between a hypothetical cause (elevated inflammatory marker) and an outcome (blood pressure) was not feasible in our cross-sectional study, because they were measured at the same time. However, we observed a potential threshold effect, as only women in the third and fourth quartile of hs-CRP and both men and women in the highest quartile of WBC concentrations exhibit significantly higher odds for high blood pressure.

The association between inflammatory markers and blood pressure may be considered plausible and coherent, as it is consistent with some of the current evidence regarding the pathogenesis of hypertension. The expression of inflammatory cytokines is induced in the early stages of hypertension, and inflammation may alter vascular function, renal blood flow, and sodium transporter expression [38–41]. Notably, while these mechanistic data link immune responses to hypertension, the role of cytokines as biomarkers of inflammation in hypertension remains poorly defined [42].

Our previous observation that canakinumab, a monoclonal antibody targeting IL-1β and reducing both IL-6 and CRP, did not reduce blood pressure or the development of hypertension in a population of CANTOS (Canakinumab Anti-Inflammatory Thrombosis Outcomes Study) trial [43] makes reverse causality less likely. Finally, there is some analogy supporting the relationship between other inflammatory markers and cardiovascular diseases. Based on human genetic evidence, IL-6 receptor–related pathways appear to have a direct causal association with coronary heart disease [15].

Some limitations of this study should be acknowledged. First, the study was based on cross-sectional data. Therefore, it is impossible to establish whether blood pressure promotes inflammation or whether inflammation precedes the development of hypertension. Most importantly, we cannot eliminate numerous potential confounders, including medications influencing both the levels of inflammatory markers and blood pressure [44]. Furthermore, our analyses are based on single hs-CRP, IL-6, and WBC measurements, which does not reflect their potential change over time. The observation of a distinct association of IL-6 with blood pressure than of hs-CRP or WBC may reflect either higher variability of IL-6 or susceptibility to confounding by other factors. IL-6 has a shorter plasma half-life than CRP, which may be a more consistent marker of chronic inflammation. To avoid confounding by infectious or inflammatory process, we evaluated only individuals without clinically significant inflammation (CRP < 10 mg/L), since the low-grade inflammatory state differs significantly from the acute inflammation [45]. The higher prevalence of male sex in the older adult group compared to the middle-aged group could be due to the study’s selective criteria, which included only participants with complete tests and excluded those with significant inflammation, potentially biasing the gender distribution. This issue was in part controlled for by performing analysis in a sex-specific manner. Therefore, the results of the study may be generalizable only to groups without clinically significant inflammation, with demographics similar to those in the PolSenior Study.

In conclusion, implementing the Bradford Hill criteria in the current epidemiologic study showed modest and inconsistent evidence for a sex-specific potential causal relationship between inflammatory markers (hs-CRP, WBC) and blood pressure. The observed association may possibly be explained by other confounding factors as well. Inflammatory markers are likely to be indirect markers of response to a variety of factors, which may influence their association with blood pressure in older adults. Whether inflammatory markers are signs of disease or causal factors for the development of hypertension remains to be determined.

## Data Availability

The data that support the findings of this study are available from the corresponding author upon reasonable request.
